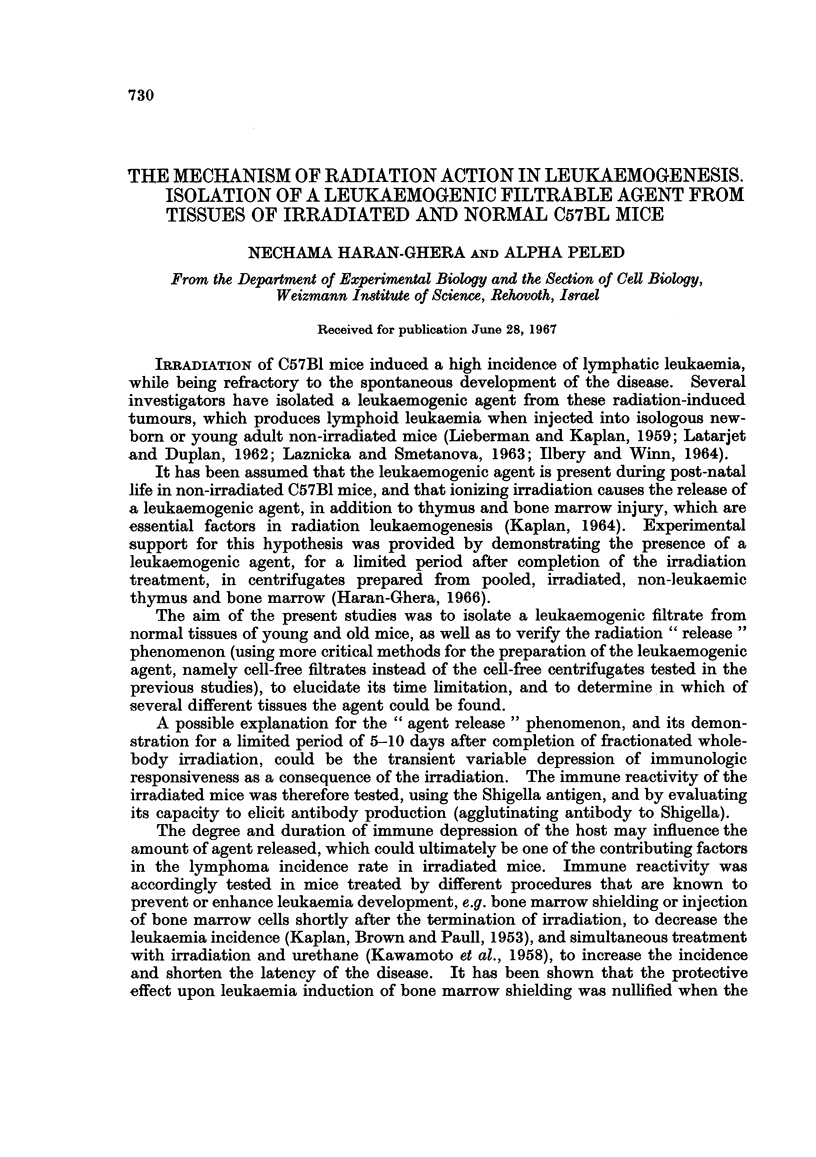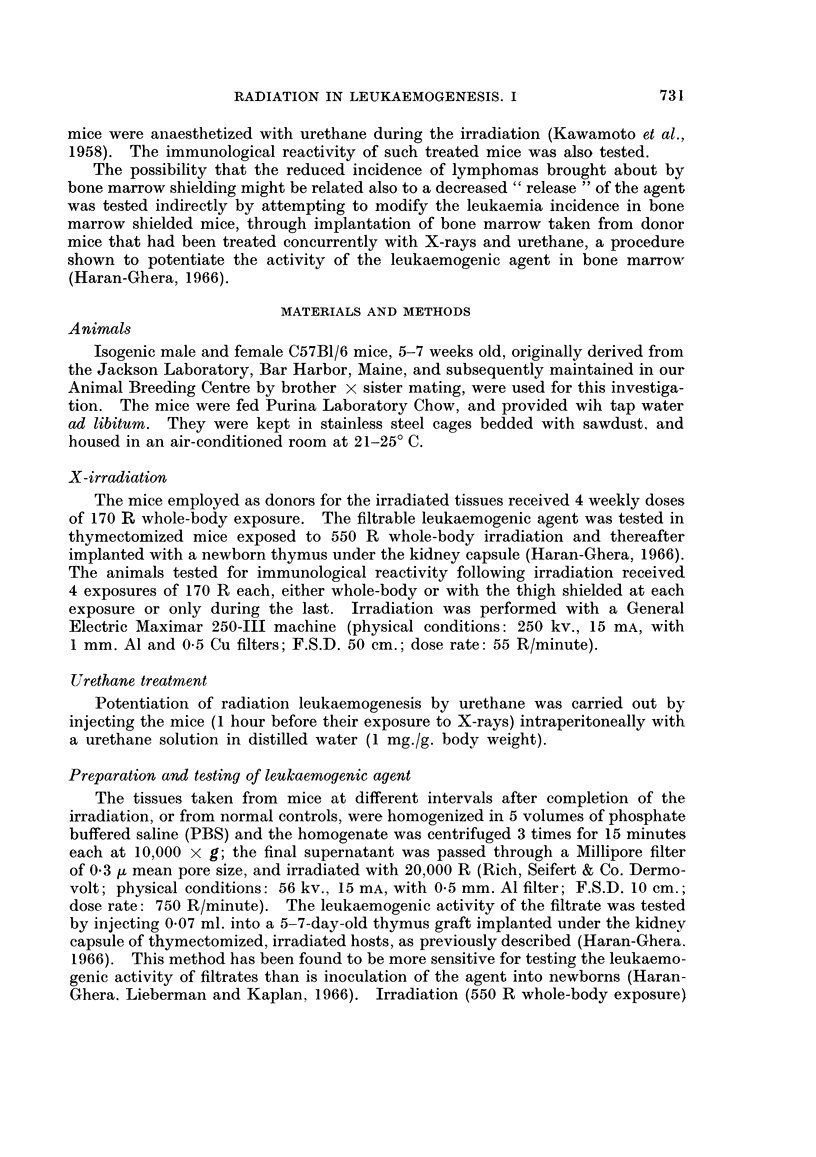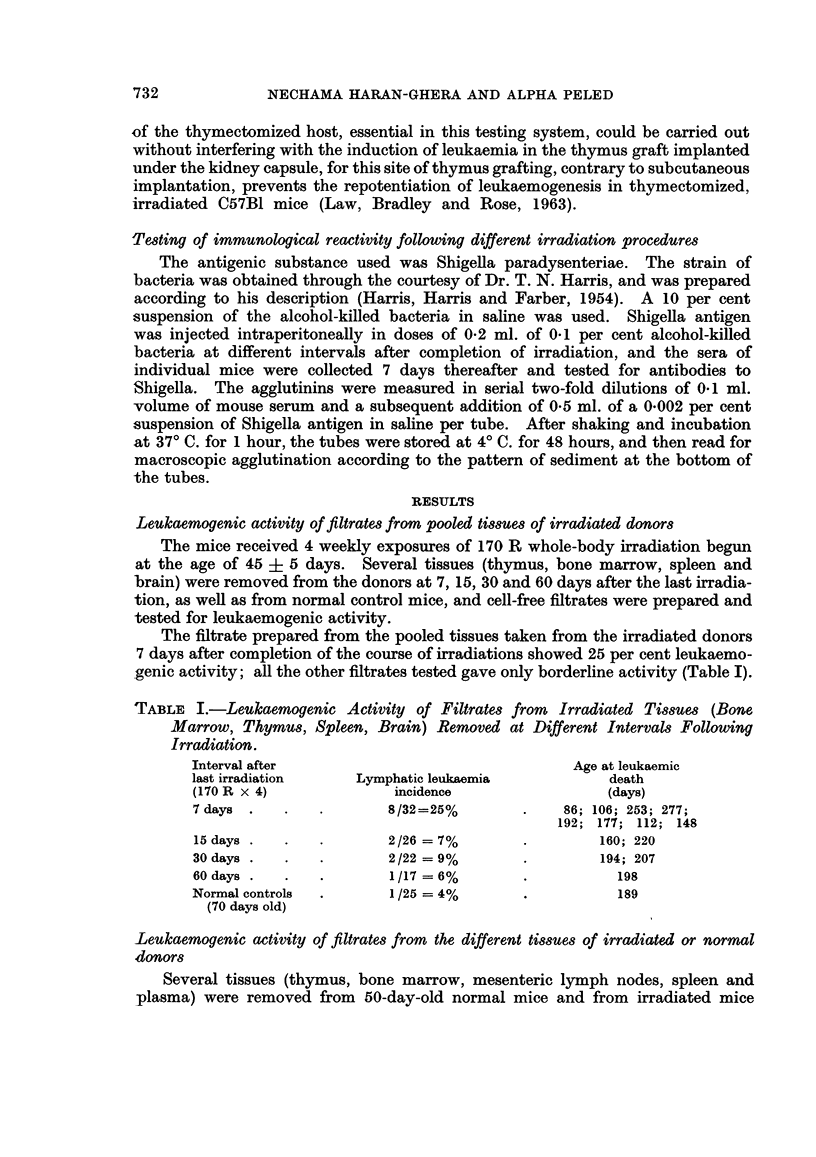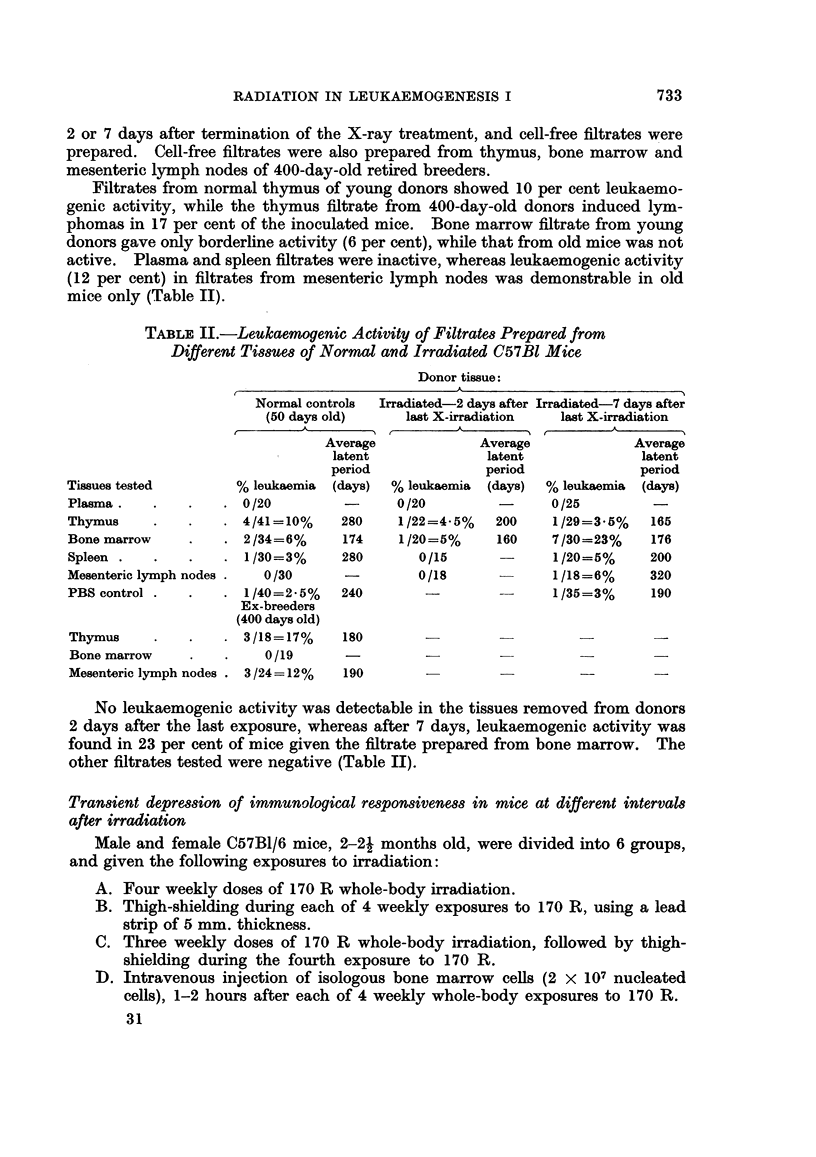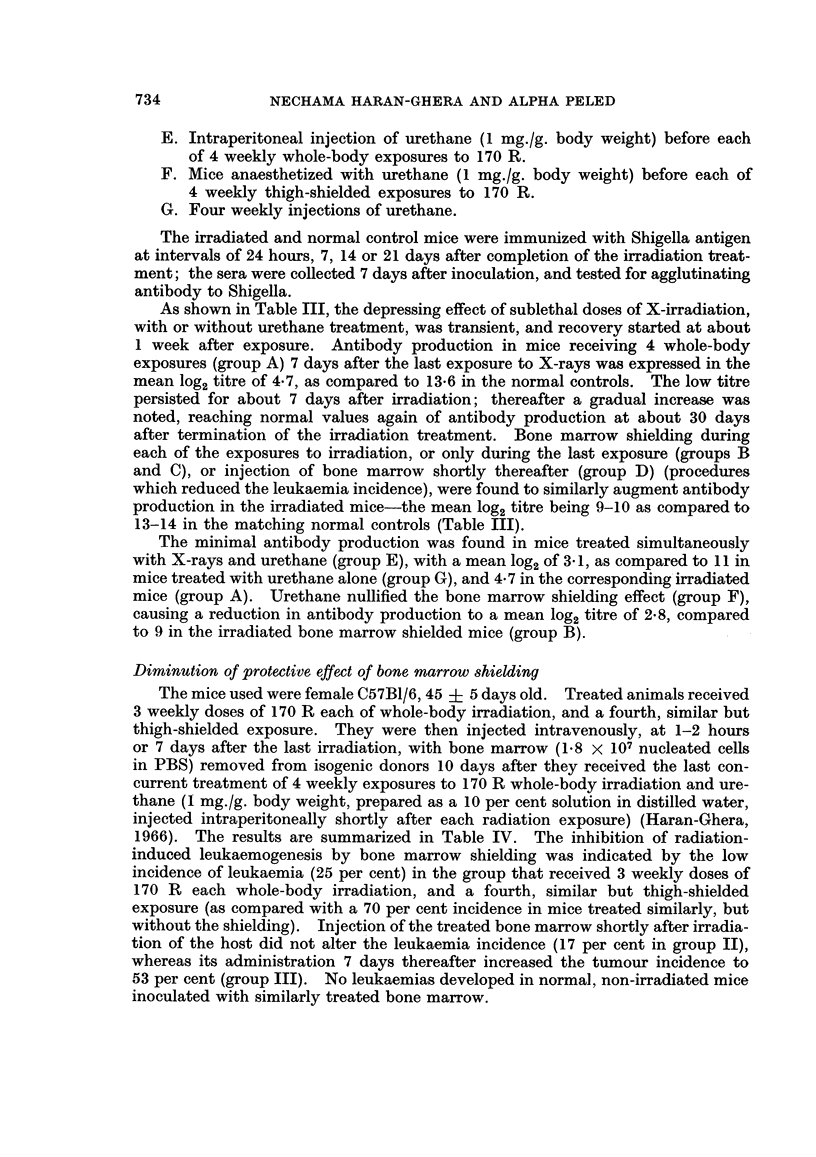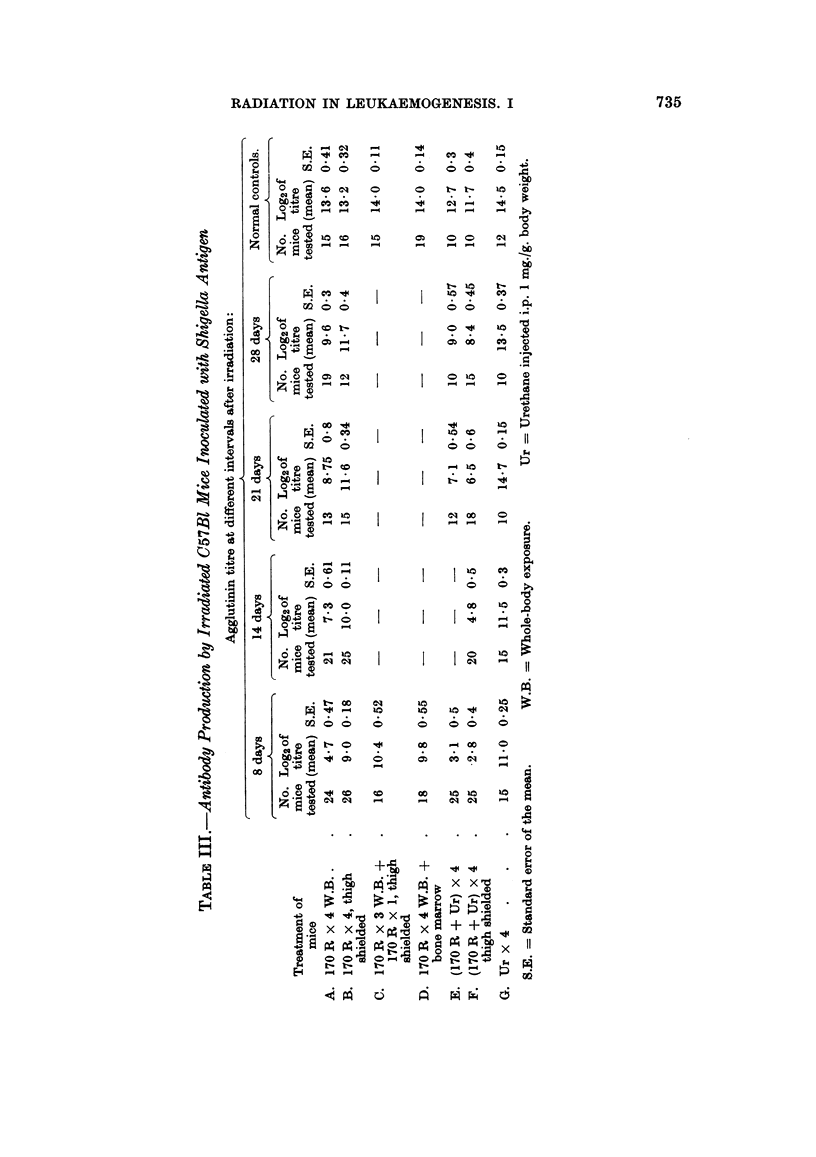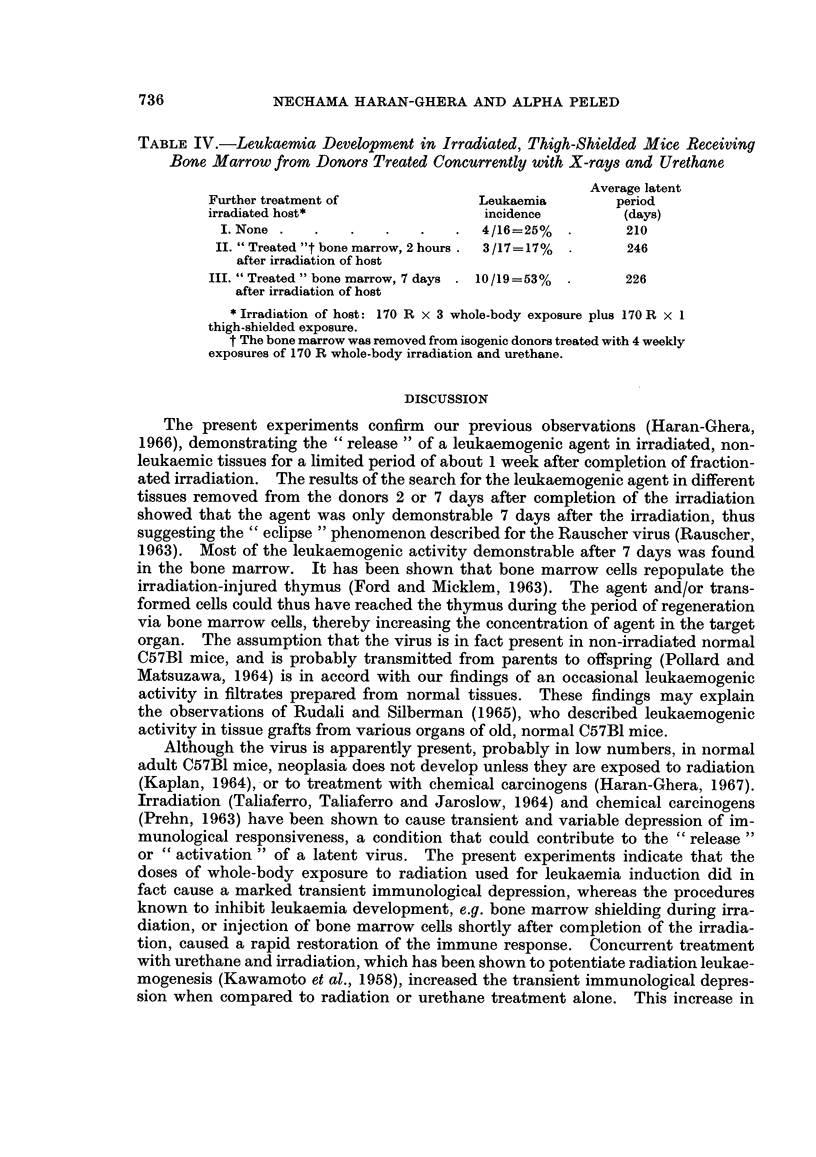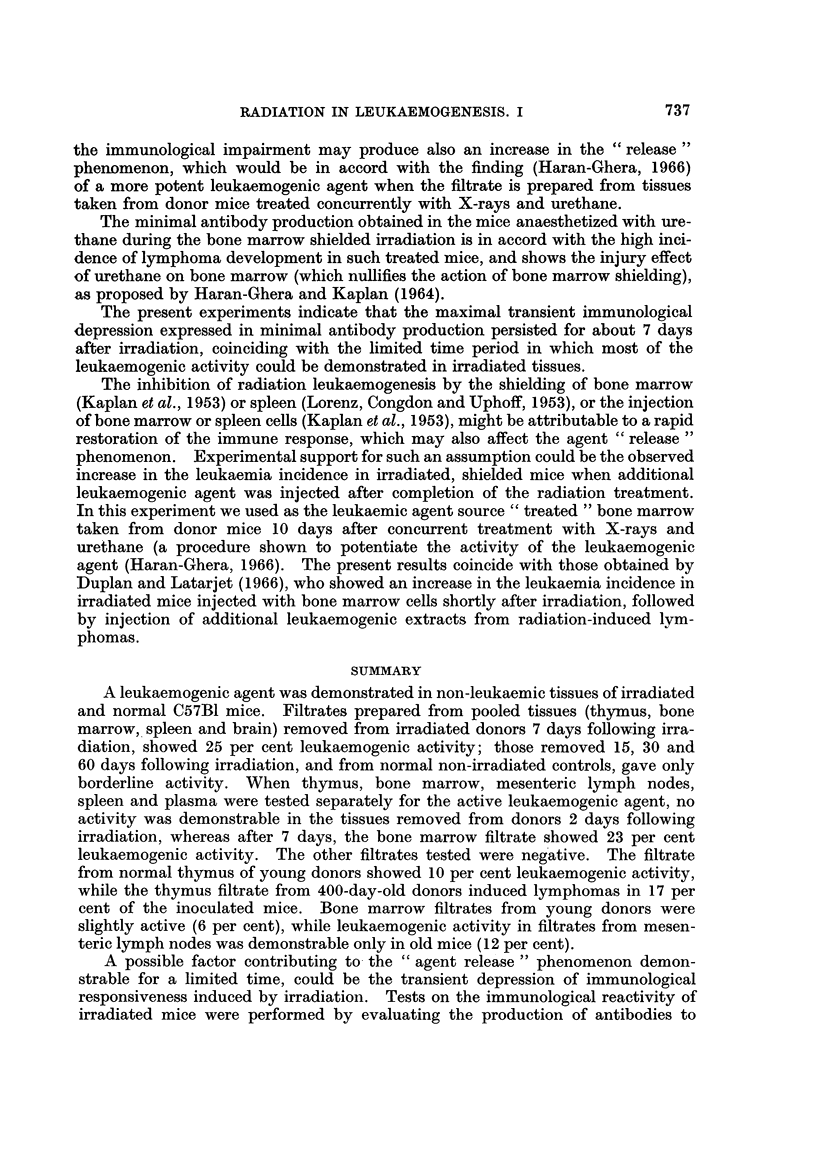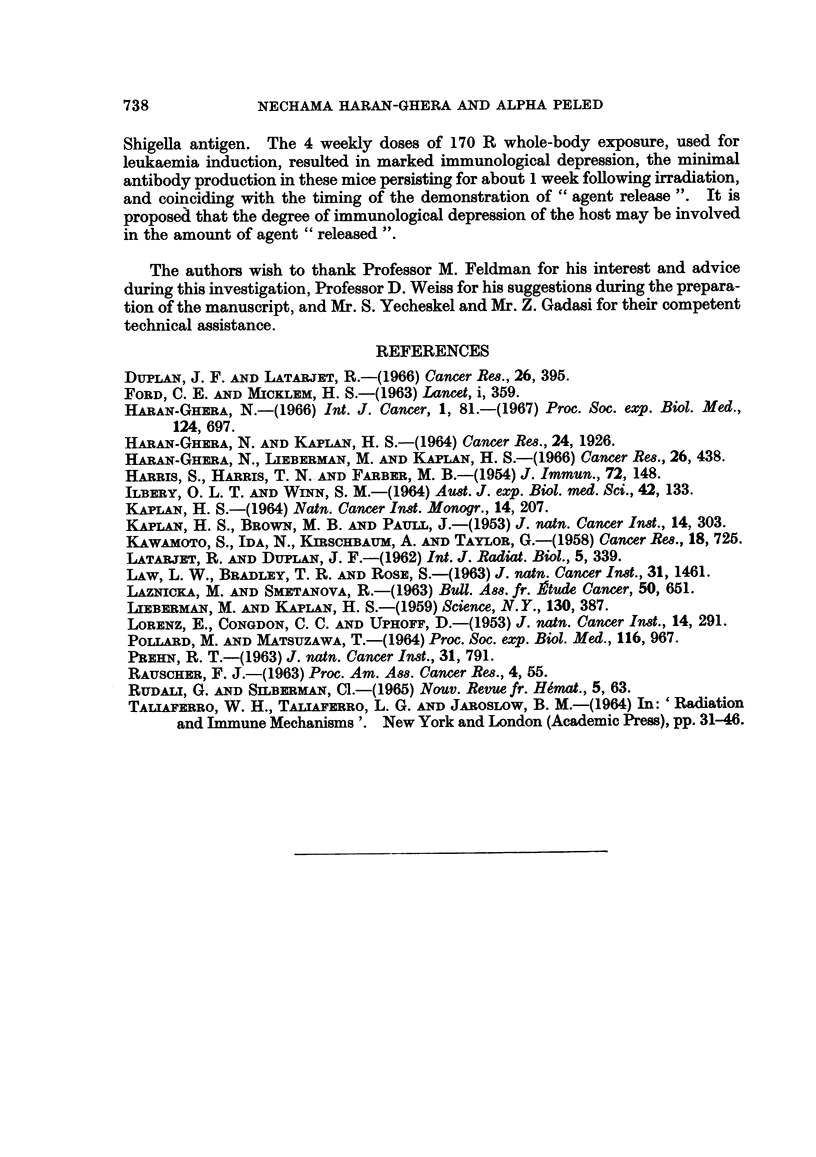# The mechanism of radiation action in leukaemogenesis. Isolation of a leukaemogenic filtrable agent from tissues of irradiated and normal C57BL mice.

**DOI:** 10.1038/bjc.1967.85

**Published:** 1967-12

**Authors:** N. Haran-Ghera, A. Peled


### THE MECHANISM OF RADIATION ACTION IN LEUKAEMOGENESIS. ISOLATION OF A LEUKAEMOGENIC FILTRABLE AGENT FROM TISSUES OF IRRADIATED AND NORMAL C57BL MICE

NECHAMA HARAN-GHERA AND ALPHA PELED

*From the Department of Experimental Biology and the Section of Cell Biology, Weizmann Institute of Science, Rehovoth, Israel*

Received for publication June 28, 1967

IRRADIATION of C57Bl mice induced a high incidence of lymphatic leukaemia, while being refractory to the spontaneous development of the disease. Several investigators have isolated a leukaemogenic agent from these radiation-induced tumours, which produces lymphoid leukaemia when injected into isologous newborn or young adult non-irradiated mice (Lieberman and Kaplan, 1959; Latarjet and Duplan, 1962; Laznicka and Smetanova, 1963; Ilbery and Winn, 1964).

It has been assumed that the leukaemogenic agent is present during post-natal life in non-irradiated C57Bl mice, and that ionizing irradiation causes the release of a leukaemogenic agent, in addition to thymus and bone marrow injury, which are essential factors in radiation leukaemogenesis (Kaplan, 1964). Experimental support for this hypothesis was provided by demonstrating the presence of a leukaemogenic agent, for a limited period after completion of the irradiation treatment, in centrifugates prepared from pooled, irradiated, non-leukaemic thymus and bone marrow (Haran-Ghera, 1966).

The aim of the present studies was to isolate a leukaemogenic filtrate from normal tissues of young and old mice, as well as to verify the radiation " release " phenomenon (using more critical methods for the preparation of the leukaemogenic agent, namely cell-free filtrates instead of the cell-free centrifugates tested in the previous studies), to elucidate its time limitation, and to determine in which of several different tissues the agent could be found.

A possible explanation for the " agent release " phenomenon, and its demonstration for a limited period of 5–10 days after completion of fractionated whole-body irradiation, could be the transient variable depression of immunologic responsiveness as a consequence of the irradiation. The immune reactivity of the irradiated mice was therefore tested, using the Shigella antigen, and by evaluating its capacity to elicit antibody production (agglutinating antibody to Shigella).

The degree and duration of immune depression of the host may influence the amount of agent released, which could ultimately be one of the contributing factors in the lymphoma incidence rate in irradiated mice. Immune reactivity was accordingly tested in mice treated by different procedures that are known to prevent or enhance leukaemia development, *e.g.* bone marrow shielding or injection of bone marrow cells shortly after the termination of irradiation, to decrease the leukaemia incidence (Kaplan, Brown and Paull, 1953), and simultaneous treatment with irradiation and urethane (Kawamoto *et al.*, 1958), to increase the incidence and shorten the latency of the disease. It has been shown that the protective effect upon leukaemia induction of bone marrow shielding was nullified when the mice were anaesthetized with urethane during the irradiation (Kawamoto *et al.*, 1958). The immunological reactivity of such treated mice was also tested.

The possibility that the reduced incidence of lymphomas brought about by bone marrow shielding might be related also to a decreased " release " of the agent was tested indirectly by attempting to modify the leukaemia incidence in bone marrow shielded mice, through implantation of bone marrow taken from donor mice that had been treated concurrently with X-rays and urethane, a procedure shown to potentiate the activity of the leukaemogenic agent in bone marrow (Haran-Ghera, 1966).

### MATERIALS AND METHODS

### *Animals*

Isogenic male and female C57Bl/6 mice, 5–7 weeks old, originally derived from the Jackson Laboratory, Bar Harbor, Maine, and subsequently maintained in our Animal Breeding Centre by brother × sister mating, were used for this investigation. The mice were fed Purina Laboratory Chow, and provided wih tap water *ad libitum*. They were kept in stainless steel cages bedded with sawdust, and housed in an air-conditioned room at 21–25° C.

### *X-irradiation*

The mice employed as donors for the irradiated tissues received 4 weekly doses of 170 R whole-body exposure. The filtrable leukaemogenic agent was tested in thymectomized mice exposed to 550 R whole-body irradiation and thereafter implanted with a newborn thymus under the kidney capsule (Haran-Ghera, 1966). The animals tested for immunological reactivity following irradiation received 4 exposures of 170 R each, either whole-body or with the thigh shielded at each exposure or only during the last. Irradiation was performed with a General Electric Maximar 250-III machine (physical conditions: 250 kv., 15 mA, with 1 mm. Al and 0·5 Cu filters; F.S.D. 50 cm.; dose rate: 55 R/minute).

### *Urethane treatment*

Potentiation of radiation leukaemogenesis by urethane was carried out by injecting the mice (1 hour before their exposure to X-rays) intraperitoneally with a urethane solution in distilled water (1 mg./g. body weight).

### *Preparation and testing of leukaemogenic agent*

The tissues taken from mice at different intervals after completion of the irradiation, or from normal controls, were homogenized in 5 volumes of phosphate buffered saline (PBS) and the homogenate was centrifuged 3 times for 15 minutes each at 10,000 × $g$; the final supernatant was passed through a Millipore filter of 0·3 $\mu$ mean pore size, and irradiated with 20,000 R (Rich, Seifert & Co. Dermovolt; physical conditions: 56 kv., 15 mA, with 0·5 mm. Al filter; F.S.D. 10 cm.; dose rate: 750 R/minute). The leukaemogenic activity of the filtrate was tested by injecting 0·07 ml. into a 5–7-day-old thymus graft implanted under the kidney capsule of thymectomized, irradiated hosts, as previously described (Haran-Ghera. 1966). This method has been found to be more sensitive for testing the leukaemogenic activity of filtrates than is inoculation of the agent into newborns (Haran-Ghera, Lieberman and Kaplan, 1966). Irradiation (550 R whole-body exposure)

of the thymectomized host, essential in this testing system, could be carried out without interfering with the induction of leukaemia in the thymus graft implanted under the kidney capsule, for this site of thymus grafting, contrary to subcutaneous implantation, prevents the repotentiation of leukaemogenesis in thymectomized, irradiated C57Bl mice (Law, Bradley and Rose, 1963).

*Testing of immunological reactivity following different irradiation procedures*

The antigenic substance used was Shigella paradysenteriae. The strain of bacteria was obtained through the courtesy of Dr. T. N. Harris, and was prepared according to his description (Harris, Harris and Farber, 1954). A 10 per cent suspension of the alcohol-killed bacteria in saline was used. Shigella antigen was injected intraperitoneally in doses of 0·2 ml. of 0·1 per cent alcohol-killed bacteria at different intervals after completion of irradiation, and the sera of individual mice were collected 7 days thereafter and tested for antibodies to Shigella. The agglutinins were measured in serial two-fold dilutions of 0·1 ml. volume of mouse serum and a subsequent addition of 0·5 ml. of a 0·002 per cent suspension of Shigella antigen in saline per tube. After shaking and incubation at 37° C. for 1 hour, the tubes were stored at 4° C. for 48 hours, and then read for macroscopic agglutination according to the pattern of sediment at the bottom of the tubes.

### RESULTS

*Leukaemogenic activity of filtrates from pooled tissues of irradiated donors*

The mice received 4 weekly exposures of 170 R whole-body irradiation begun at the age of 45 $\pm$ 5 days. Several tissues (thymus, bone marrow, spleen and brain) were removed from the donors at 7, 15, 30 and 60 days after the last irradiation, as well as from normal control mice, and cell-free filtrates were prepared and tested for leukaemogenic activity.

The filtrate prepared from the pooled tissues taken from the irradiated donors 7 days after completion of the course of irradiations showed 25 per cent leukaemogenic activity; all the other filtrates tested gave only borderline activity (Table I).

TABLE I.—*Leukaemogenic Activity of Filtrates from Irradiated Tissues (Bone Marrow, Thymus, Spleen, Brain) Removed at Different Intervals Following Irradiation.*

| Interval after last irradiation (170 R × 4) | Lymphatic leukaemia incidence | Age at leukaemic death (days) |
|---|---|---|
| 7 days | 8/32=25% | 86; 106; 253; 277; 192; 177; 112; 148 |
| 15 days | 2/26 = 7% | 160; 220 |
| 30 days | 2/22 = 9% | 194; 207 |
| 60 days | 1/17 = 6% | 198 |
| Normal controls (70 days old) | 1/25 = 4% | 189 |

*Leukaemogenic activity of filtrates from the different tissues of irradiated or normal donors*

Several tissues (thymus, bone marrow, mesenteric lymph nodes, spleen and plasma) were removed from 50-day-old normal mice and from irradiated mice

2 or 7 days after termination of the X-ray treatment, and cell-free filtrates were prepared. Cell-free filtrates were also prepared from thymus, bone marrow and mesenteric lymph nodes of 400-day-old retired breeders.

Filtrates from normal thymus of young donors showed 10 per cent leukaemogenic activity, while the thymus filtrate from 400-day-old donors induced lymphomas in 17 per cent of the inoculated mice. Bone marrow filtrate from young donors gave only borderline activity (6 per cent), while that from old mice was not active. Plasma and spleen filtrates were inactive, whereas leukaemogenic activity (12 per cent) in filtrates from mesenteric lymph nodes was demonstrable in old mice only (Table II).

TABLE II.—*Leukaemogenic Activity of Filtrates Prepared from Different Tissues of Normal and Irradiated C57Bl Mice*

| | Donor tissue: Normal controls (50 days old) | | Irradiated—2 days after last X-irradiation | | Irradiated—7 days after last X-irradiation | |
|---|---|---|---|---|---|---|
| Tissues tested | % leukaemia | Average latent period (days) | % leukaemia | Average latent period (days) | % leukaemia | Average latent period (days) |
| Plasma . . . . | 0/20 | — | 0/20 | — | 0/25 | — |
| Thymus . . . | 4/41=10% | 280 | 1/22=4·5% | 200 | 1/29=3·5% | 165 |
| Bone marrow . . | 2/34=6% | 174 | 1/20=5% | 160 | 7/30=23% | 176 |
| Spleen . . . . | 1/30=3% | 280 | 0/15 | — | 1/20=5% | 200 |
| Mesenteric lymph nodes . | 0/30 | — | 0/18 | — | 1/18=6% | 320 |
| PBS control . . . | 1/40=2·5% | 240 | — | — | 1/35=3% | 190 |
| | Ex-breeders (400 days old) | | | | | |
| Thymus . . . | 3/18=17% | 180 | — | — | — | — |
| Bone marrow . . | 0/19 | — | — | — | — | — |
| Mesenteric lymph nodes . | 3/24=12% | 190 | — | — | — | — |

No leukaemogenic activity was detectable in the tissues removed from donors 2 days after the last exposure, whereas after 7 days, leukaemogenic activity was found in 23 per cent of mice given the filtrate prepared from bone marrow. The other filtrates tested were negative (Table II).

*Transient depression of immunological responsiveness in mice at different intervals after irradiation*

Male and female C57Bl/6 mice, 2–2½ months old, were divided into 6 groups, and given the following exposures to irradiation:

A. Four weekly doses of 170 R whole-body irradiation.
B. Thigh-shielding during each of 4 weekly exposures to 170 R, using a lead strip of 5 mm. thickness.
C. Three weekly doses of 170 R whole-body irradiation, followed by thigh-shielding during the fourth exposure to 170 R.
D. Intravenous injection of isologous bone marrow cells ($2 \times 10^7$ nucleated cells), 1–2 hours after each of 4 weekly whole-body exposures to 170 R.

E. Intraperitoneal injection of urethane (1 mg./g. body weight) before each of 4 weekly whole-body exposures to 170 R.
F. Mice anaesthetized with urethane (1 mg./g. body weight) before each of 4 weekly thigh-shielded exposures to 170 R.
G. Four weekly injections of urethane.

The irradiated and normal control mice were immunized with Shigella antigen at intervals of 24 hours, 7, 14 or 21 days after completion of the irradiation treatment; the sera were collected 7 days after inoculation, and tested for agglutinating antibody to Shigella.

As shown in Table III, the depressing effect of sublethal doses of X-irradiation, with or without urethane treatment, was transient, and recovery started at about 1 week after exposure. Antibody production in mice receiving 4 whole-body exposures (group A) 7 days after the last exposure to X-rays was expressed in the mean $\log_2$ titre of 4·7, as compared to 13·6 in the normal controls. The low titre persisted for about 7 days after irradiation; thereafter a gradual increase was noted, reaching normal values again of antibody production at about 30 days after termination of the irradiation treatment. Bone marrow shielding during each of the exposures to irradiation, or only during the last exposure (groups B and C), or injection of bone marrow shortly thereafter (group D) (procedures which reduced the leukaemia incidence), were found to similarly augment antibody production in the irradiated mice—the mean $\log_2$ titre being 9–10 as compared to 13–14 in the matching normal controls (Table III).

The minimal antibody production was found in mice treated simultaneously with X-rays and urethane (group E), with a mean $\log_2$ of 3·1, as compared to 11 in mice treated with urethane alone (group G), and 4·7 in the corresponding irradiated mice (group A). Urethane nullified the bone marrow shielding effect (group F), causing a reduction in antibody production to a mean $\log_2$ titre of 2·8, compared to 9 in the irradiated bone marrow shielded mice (group B).

*Diminution of protective effect of bone marrow shielding*

The mice used were female C57Bl/6, 45 $\pm$ 5 days old. Treated animals received 3 weekly doses of 170 R each of whole-body irradiation, and a fourth, similar but thigh-shielded exposure. They were then injected intravenously, at 1–2 hours or 7 days after the last irradiation, with bone marrow ($1{\cdot}8 \times 10^7$ nucleated cells in PBS) removed from isogenic donors 10 days after they received the last concurrent treatment of 4 weekly exposures to 170 R whole-body irradiation and urethane (1 mg./g. body weight, prepared as a 10 per cent solution in distilled water, injected intraperitoneally shortly after each radiation exposure) (Haran-Ghera, 1966). The results are summarized in Table IV. The inhibition of radiation-induced leukaemogenesis by bone marrow shielding was indicated by the low incidence of leukaemia (25 per cent) in the group that received 3 weekly doses of 170 R each whole-body irradiation, and a fourth, similar but thigh-shielded exposure (as compared with a 70 per cent incidence in mice treated similarly, but without the shielding). Injection of the treated bone marrow shortly after irradiation of the host did not alter the leukaemia incidence (17 per cent in group II), whereas its administration 7 days thereafter increased the tumour incidence to 53 per cent (group III). No leukaemias developed in normal, non-irradiated mice inoculated with similarly treated bone marrow.

TABLE III.—*Antibody Production by Irradiated C57Bl Mice Inoculated with Shigella Antigen*

| Treatment of mice | Agglutinin titre at different intervals after irradiation: 8 days — No. mice tested | 8 days — $Log_2$ of titre (mean) | 8 days — S.E. | 14 days — No. mice tested | 14 days — $Log_2$ of titre (mean) | 14 days — S.E. | 21 days — No. mice tested | 21 days — $Log_2$ of titre (mean) | 21 days — S.E. | 28 days — No. mice tested | 28 days — $Log_2$ of titre (mean) | 28 days — S.E. | Normal controls. — No. mice tested | Normal controls. — $Log_2$ of titre (mean) | Normal controls. — S.E. |
|---|---|---|---|---|---|---|---|---|---|---|---|---|---|---|---|
| A. 170 R × 4 W.B. . | 24 | 4·7 | 0·47 | 21 | 7·3 | 0·61 | 13 | 8·75 | 0·8 | 19 | 9·6 | 0·3 | 15 | 13·6 | 0·41 |
| B. 170 R × 4, thigh shielded | 26 | 9·0 | 0·18 | 25 | 10·0 | 0·11 | 15 | 11·6 | 0·34 | 12 | 11·7 | 0·4 | 16 | 13·2 | 0·32 |
| C. 170 R × 3 W.B. + 170 R × 1, thigh shielded | 16 | 10·4 | 0·52 | — | — | — | — | — | — | — | — | — | 15 | 14·0 | 0·11 |
| D. 170 R × 4 W.B. + bone marrow | 18 | 9·8 | 0·55 | — | — | — | — | — | — | — | — | — | 19 | 14·0 | 0·14 |
| E. (170 R + Ur) × 4 | 25 | 3·1 | 0·5 | — | — | — | 12 | 7·1 | 0·54 | 10 | 9·0 | 0·57 | 10 | 12·7 | 0·3 |
| F. (170 R + Ur) × 4 thigh shielded | 25 | 2·8 | 0·4 | 20 | 4·8 | 0·5 | 18 | 6·5 | 0·6 | 15 | 8·4 | 0·45 | 10 | 11·7 | 0·4 |
| G. Ur × 4 . . . | 15 | 11·0 | 0·25 | 15 | 11·5 | 0·3 | 10 | 14·7 | 0·15 | 10 | 13·5 | 0·37 | 12 | 14·5 | 0·15 |

S.E. = Standard error of the mean. W.B. = Whole-body exposure. Ur = Urethane injected i.p. 1 mg./g. body weight.

TABLE IV.—*Leukaemia Development in Irradiated, Thigh-Shielded Mice Receiving Bone Marrow from Donors Treated Concurrently with X-rays and Urethane*

| Further treatment of irradiated host* | Leukaemia incidence | Average latent period (days) |
|---|---|---|
| I. None . . . . . . . | 4/16=25% . | 210 |
| II. " Treated "† bone marrow, 2 hours after irradiation of host . | 3/17=17% . | 246 |
| III. " Treated " bone marrow, 7 days after irradiation of host . | 10/19=53% . | 226 |

* Irradiation of host: 170 R × 3 whole-body exposure plus 170 R × 1 thigh-shielded exposure.

† The bone marrow was removed from isogenic donors treated with 4 weekly exposures of 170 R whole-body irradiation and urethane.

### DISCUSSION

The present experiments confirm our previous observations (Haran-Ghera, 1966), demonstrating the " release " of a leukaemogenic agent in irradiated, non-leukaemic tissues for a limited period of about 1 week after completion of fractionated irradiation. The results of the search for the leukaemogenic agent in different tissues removed from the donors 2 or 7 days after completion of the irradiation showed that the agent was only demonstrable 7 days after the irradiation, thus suggesting the " eclipse " phenomenon described for the Rauscher virus (Rauscher, 1963). Most of the leukaemogenic activity demonstrable after 7 days was found in the bone marrow. It has been shown that bone marrow cells repopulate the irradiation-injured thymus (Ford and Micklem, 1963). The agent and/or transformed cells could thus have reached the thymus during the period of regeneration via bone marrow cells, thereby increasing the concentration of agent in the target organ. The assumption that the virus is in fact present in non-irradiated normal C57Bl mice, and is probably transmitted from parents to offspring (Pollard and Matsuzawa, 1964) is in accord with our findings of an occasional leukaemogenic activity in filtrates prepared from normal tissues. These findings may explain the observations of Rudali and Silberman (1965), who described leukaemogenic activity in tissue grafts from various organs of old, normal C57Bl mice.

Although the virus is apparently present, probably in low numbers, in normal adult C57Bl mice, neoplasia does not develop unless they are exposed to radiation (Kaplan, 1964), or to treatment with chemical carcinogens (Haran-Ghera, 1967). Irradiation (Taliaferro, Taliaferro and Jaroslow, 1964) and chemical carcinogens (Prehn, 1963) have been shown to cause transient and variable depression of immunological responsiveness, a condition that could contribute to the " release " or " activation " of a latent virus. The present experiments indicate that the doses of whole-body exposure to radiation used for leukaemia induction did in fact cause a marked transient immunological depression, whereas the procedures known to inhibit leukaemia development, *e.g.* bone marrow shielding during irradiation, or injection of bone marrow cells shortly after completion of the irradiation, caused a rapid restoration of the immune response. Concurrent treatment with urethane and irradiation, which has been shown to potentiate radiation leukaemogenesis (Kawamoto *et al.*, 1958), increased the transient immunological depression when compared to radiation or urethane treatment alone. This increase in the immunological impairment may produce also an increase in the " release " phenomenon, which would be in accord with the finding (Haran-Ghera, 1966) of a more potent leukaemogenic agent when the filtrate is prepared from tissues taken from donor mice treated concurrently with X-rays and urethane.

The minimal antibody production obtained in the mice anaesthetized with urethane during the bone marrow shielded irradiation is in accord with the high incidence of lymphoma development in such treated mice, and shows the injury effect of urethane on bone marrow (which nullifies the action of bone marrow shielding), as proposed by Haran-Ghera and Kaplan (1964).

The present experiments indicate that the maximal transient immunological depression expressed in minimal antibody production persisted for about 7 days after irradiation, coinciding with the limited time period in which most of the leukaemogenic activity could be demonstrated in irradiated tissues.

The inhibition of radiation leukaemogenesis by the shielding of bone marrow (Kaplan *et al.*, 1953) or spleen (Lorenz, Congdon and Uphoff, 1953), or the injection of bone marrow or spleen cells (Kaplan *et al.*, 1953), might be attributable to a rapid restoration of the immune response, which may also affect the agent " release " phenomenon. Experimental support for such an assumption could be the observed increase in the leukaemia incidence in irradiated, shielded mice when additional leukaemogenic agent was injected after completion of the radiation treatment. In this experiment we used as the leukaemic agent source " treated " bone marrow taken from donor mice 10 days after concurrent treatment with X-rays and urethane (a procedure shown to potentiate the activity of the leukaemogenic agent (Haran-Ghera, 1966). The present results coincide with those obtained by Duplan and Latarjet (1966), who showed an increase in the leukaemia incidence in irradiated mice injected with bone marrow cells shortly after irradiation, followed by injection of additional leukaemogenic extracts from radiation-induced lymphomas.

### SUMMARY

A leukaemogenic agent was demonstrated in non-leukaemic tissues of irradiated and normal C57Bl mice. Filtrates prepared from pooled tissues (thymus, bone marrow, spleen and brain) removed from irradiated donors 7 days following irradiation, showed 25 per cent leukaemogenic activity; those removed 15, 30 and 60 days following irradiation, and from normal non-irradiated controls, gave only borderline activity. When thymus, bone marrow, mesenteric lymph nodes, spleen and plasma were tested separately for the active leukaemogenic agent, no activity was demonstrable in the tissues removed from donors 2 days following irradiation, whereas after 7 days, the bone marrow filtrate showed 23 per cent leukaemogenic activity. The other filtrates tested were negative. The filtrate from normal thymus of young donors showed 10 per cent leukaemogenic activity, while the thymus filtrate from 400-day-old donors induced lymphomas in 17 per cent of the inoculated mice. Bone marrow filtrates from young donors were slightly active (6 per cent), while leukaemogenic activity in filtrates from mesenteric lymph nodes was demonstrable only in old mice (12 per cent).

A possible factor contributing to the " agent release " phenomenon demonstrable for a limited time, could be the transient depression of immunological responsiveness induced by irradiation. Tests on the immunological reactivity of irradiated mice were performed by evaluating the production of antibodies to

Shigella antigen. The 4 weekly doses of 170 R whole-body exposure, used for leukaemia induction, resulted in marked immunological depression, the minimal antibody production in these mice persisting for about 1 week following irradiation, and coinciding with the timing of the demonstration of " agent release ". It is proposed that the degree of immunological depression of the host may be involved in the amount of agent " released ".

The authors wish to thank Professor M. Feldman for his interest and advice during this investigation, Professor D. Weiss for his suggestions during the preparation of the manuscript, and Mr. S. Yecheskel and Mr. Z. Gadasi for their competent technical assistance.

### REFERENCES

DUPLAN, J. F. AND LATARJET, R.—(1966) *Cancer Res.*, **26**, 395.

FORD, C. E. AND MICKLEM, H. S.—(1963) *Lancet*, i, 359.

HARAN-GHERA, N.—(1966) *Int. J. Cancer*, **1**, 81.—(1967) *Proc. Soc. exp. Biol. Med.*, **124**, 697.

HARAN-GHERA, N. AND KAPLAN, H. S.—(1964) *Cancer Res.*, **24**, 1926.

HARAN-GHERA, N., LIEBERMAN, M. AND KAPLAN, H. S.—(1966) *Cancer Res.*, **26**, 438.

HARRIS, S., HARRIS, T. N. AND FARBER, M. B.—(1954) *J. Immun.*, **72**, 148.

ILBERY, O. L. T. AND WINN, S. M.—(1964) *Aust. J. exp. Biol. med. Sci.*, **42**, 133.

KAPLAN, H. S.—(1964) *Natn. Cancer Inst. Monogr.*, **14**, 207.

KAPLAN, H. S., BROWN, M. B. AND PAULL, J.—(1953) *J. natn. Cancer Inst.*, **14**, 303.

KAWAMOTO, S., IDA, N., KIRSCHBAUM, A. AND TAYLOR, G.—(1958) *Cancer Res.*, **18**, 725.

LATARJET, R. AND DUPLAN, J. F.—(1962) *Int. J. Radiat. Biol.*, **5**, 339.

LAW, L. W., BRADLEY, T. R. AND ROSE, S.—(1963) *J. natn. Cancer Inst.*, **31**, 1461.

LAZNICKA, M. AND SMETANOVA, R.—(1963) *Bull. Ass. fr. Étude Cancer*, **50**, 651.

LIEBERMAN, M. AND KAPLAN, H. S.—(1959) *Science, N.Y.*, **130**, 387.

LORENZ, E., CONGDON, C. C. AND UPHOFF, D.—(1953) *J. natn. Cancer Inst.*, **14**, 291.

POLLARD, M. AND MATSUZAWA, T.—(1964) *Proc. Soc. exp. Biol. Med.*, **116**, 967.

PREHN, R. T.—(1963) *J. natn. Cancer Inst.*, **31**, 791.

RAUSCHER, F. J.—(1963) *Proc. Am. Ass. Cancer Res.*, **4**, 55.

RUDALI, G. AND SILBERMAN, Cl.—(1965) *Nouv. Revue fr. Hémat.*, **5**, 63.

TALIAFERRO, W. H., TALIAFERRO, L. G. AND JAROSLOW, B. M.—(1964) In: ' Radiation and Immune Mechanisms '. New York and London (Academic Press), pp. 31–46.